# A comparative appraisal of stigma among healthcare workers towards alcohol and substance use disorders: a case vignette study

**DOI:** 10.3389/fpsyt.2026.1634817

**Published:** 2026-02-18

**Authors:** Onat Yilmaz, Alparslan Asil Budakli, Eda Aslan

**Affiliations:** 1 Department of Psychiatry, Okmeydani Education and Research Hospital, Istanbul, Türkiye; 2 Department of Psychiatry, Bahcesehir University Faculty of Medicine, Kadikoy, Istanbul, Türkiye; 3 Department of Psychiatry, Mersin University Faculty of Medicine, Ciftlikkoy, Mersin, Türkiye

**Keywords:** stigma, alcohol use disorder, substance use disorder, healthcare, healthcare workers

## Abstract

**Introduction:**

Alcohol and substance use disorders contribute significantly to the global burden of disease, resulting in substantial morbidity and mortality. Meanwhile, stigmatization is a major barrier to treatment and incorporation into community life processes. Given that stigma among health care workers may affect patients’ access to adequate healthcare services, this study aimed to evaluate the stigma patterns regarding alcohol and substance use disorders.

**Materials and Methods:**

This descriptive study was conducted at a tertiary health facility. Two identical case vignettes were prepared to evaluate the possible differences in stigma patterns of alcohol and substance use disorders. The target population included physicians, nurses, and other healthcare workers. A total of 396 voluntary participants completed the study.

**Results:**

The proportion of participants who stated that substance use disorder was a more severe illness was significantly higher, while alcohol use disorder was considered to be more associated with socioeconomic problems. Similarly, participants felt that substance use patients should not freely roam in the community, were more aggressive, and cannot make sound decisions in life. Finally, patients with substance use disorders were seen as unable to recover completely.

**Discussion:**

To the best of our knowledge, this is the first study to evaluate differences of stigmatization patterns regarding alcohol and substance use disorders among healthcare workers. The study showed that the stigma associated with alcohol and substance use disorders was high, while substance use was also associated with higher levels of unacceptability and greater social distancing. These findings can be used in developing health policies and enhancing educational strategies.

## 1 Introduction

Mental disorders have been associated with the burden of disease globally. Among them, substance/alcohol use disorders have devastating effects on both individuals and society. Hence, many national and international policymakers have taken action on different topics, and the World Health Organization (WHO) has implemented a Special Initiative for Mental Health, targeting to improve access to mental health services and diminish health disparities among those affected (1). WHO reported that approximately 43% of the world’s population over 15 years of age currently drinks alcohol, and the highest prevalence of alcohol consumption is observed in the WHO-European Region, of which approximately 60% of drinkers are categorized as heavy drinkers. This pattern of alcohol consumption accounts for 10.1% of all deaths and 10.8% of all disability-adjusted life-years (DALYs) in the region (2). Additionally, the same report indicated that approximately 5.5% of the global population between 15 to 64 years of age used various substances in the previous year, and approximately 35 million of them met the criteria for any substance use disorder (2). The global increase in the number of substance/alcohol use disorders has made the management and prevention of these disorders a primary concern in society.

Stigma was initially described by Goffman (3), and has been a topic of interest for researchers because of its consequences, including decreased social interaction, decreased treatment adherence, and hospital admission (3, 4). The concept of stigma has been determined in terms of prejudice, discrimination, labelling, loss of status, and stereotyping, and has been recognized at structural (such as regulations, laws, etc.), interpersonal (such as beliefs, attitudes, etc.), and intrapersonal (self-stigma) levels (5). Mental disorders are among the most striking examples of clinical conditions in which patients face stigmatization, and various patterns of stigmatization have been commonly described in cases of alcohol and substance use disorders (ASUD) (6).

Research in population settings addresses various causal etiological perceptions of ASUD, including, but not limited to, character traits, economic difficulties, emotional or physical traumas, and illegal activities (7). Studies indicate that the stigmatization of patients by health professionals may have unfavorable effects on therapeutic relationships, treatment referral, treatment adherence, and prognosis (8).

Almost all of the substances listed in classification systems are prohibited from being used, traded, or possessed in many countries, and legal sanctions are imposed on individuals engaging in such acts. Nevertheless, selling, supplying and consumption of alcohol are regulated rather than prohibited by law (9). In their article, Corrigan and colleagues described alcohol and tobacco as “socially benign substances” and emphasized that individuals subjected to legal sanctions due to substance use or trade, experience significantly higher levels of structural stigma (10). Additionally, there is limited data evaluating the differences between alcohol and other substance use disorders in the context of stigmatization. Although several hypotheses have been proposed regarding differences in the stigmatization of individuals diagnosed with AUD versus SUD, no study to date has directly investigated this subject among healthcare professionals. The available data have been inferred from studies conducted on patients with ASUD.

Thus, evaluating the stigma towards ASUD among health professionals is crucial in setting up preventive measures to maximize patient care. Since there is limited data in the literature, research on this topic may reveal unidentified stigma domains, and help develop an example for healthcare providers. To the best of our knowledge, this is the first study to assess and compare the stigma patterns of alcohol and substance use disorders in a tertiary healthcare center with the participation of healthcare workers in varying occupational positions.

## 2 Materials and methods

### 2.1 Setting and participants

This study was designed as a descriptive study and conducted at the Okmeydani Education and Research Hospital, a medical center with one of the highest outpatient volumes in Istanbul. Medical staff at the hospital were invited to participate in the study; the case vignettes were distributed among 428 personnel (38 physicians, 144 nurses, and 246 other health workers-secretaries and caregivers). The participation rate was 93%, and 396 participants completed the study. Ethical committee approval for this study was obtained from Okmeydani Education and Research Hospital (protocol number 17/604). Written informed consent was obtained from all the participants.

### 2.2 Data collection

Being over 18 years old and currently working at the health facility and heaving no history of ASUD diagnosis were the baseline requirements for participating in the study. At the beginning of the research interview, participants were briefly informed by one of the authors about the purpose, design, and potential targets of the study. Since vignettes are practical in conceptualizing the research topic, and a common method used in mental health-related studies, the methodology for the development of data collection tools and case vignettes was based on a previous reference study evaluating the stigma patterns surrounding mental disorders named the Investigation of Public Attitude for Mental Illnesses project (11). The first part of the questionnaire consisted of ten questions to evaluate participants’ sociodemographic background, including age, gender, years of education, and profession. Participants were also asked whether they had a history of mental illness, a relative with mental illness, any formal education about mental health, or previous work experience in a mental health facility. The second part included 33 questions about two case vignettes describing two hypothetical persons with characteristics matching those found in the Diagnostic and Statistical Manual of Mental Disorders, Fifth Edition (DSM-5), criteria for alcohol and substance use disorders (12). Neither the diagnoses of each vignette were shared with the participants, nor were they informed of the clinical conditions of each case.

For each hypothetical case, the first 29 statements evaluated whether the participants would like to interact with the patient, their etiological attributions about each scenario, their comments about referring cases to treatment or not, perceptions about treatment methods, and their understanding of each case. Every statement was rated by the participants in accordance with their agreement levels, from “strongly agree” to “strongly disagree.” The last four questions evaluated which specialist the participant would refer to the case, and which specialist the participant would apply if similar symptoms occurred (more details can be found in the supplementary material).

### 2.3 Case scenarios

The case vignettes, developed in accordance with the diagnostic criteria outlined in the DSM-5, were identical in structure, except for the sections describing the disorder, as detailed below:

Mrs. A, 27 years old, married, has two children, a housewife. Her husband is a factory laborer. Mrs. A frequently consumes (alcohol/substances like marijuana, cannabis, opioid or cocaine) more than or longer than expected. Mrs A constantly has a desire or inconclusive attempts to give-up or to control this consumption. Mrs A also experiences social or interpersonal problems due to using (alcohol/substances like marijuana, cannabis, opioid or cocaine), but continues to consume (alcohol/substances like marijuana, cannabis, opioid or cocaine).

### 2.4 Statistical analyses

Descriptive statistics were presented using frequency and percentage for categorical variables. Statistical comparisons of categorical variables between the independent groups formed by the two different case scenarios were performed using the chi-square test. The statistical significance was considered as a Type-I error level of 5% (p<0.05). All statistical analyses were performed using SPSS version 25 (IBM Corp., Armonk, NY, USA).

## 3 Results

A total of 396 participants were included in the study, of whom 28.8% were males (n=114) and 71.2% were females (n=282). More than half of the participants were between 18 and 25 years of age (n=226, 57.1%), while 21% were between 26 and 35 years of age (n=83). Approximately one-third of the study population was married (n=125, 31.6%), with the majority of them graduating from high school (n=244, 61.6%). As for occupation, 6.3% of the participants were doctors (n=25), 33.6% nurses (n=133), and 60.1% other allied health personnel (n=238). The frequency of history of any psychiatric treatment in the study group was 5.3% (n=21), and about one-fifth of the participants had a relative with a mental illness (n=85, 21.5%); the proportion of first-degree relatives (parent, sibling, child, spouses) among them was 39.3% (n=33). The most frequent diagnosis among participants was depression (n=32; 38.1%). The primary source for gaining knowledge about the psychiatric disorder in oneself or a relative was education in 27.5% of the participants (n=25), while 52.7% of them (n=48) gained information from a source other than television, social media, or the Internet. Eighty-four participants (21.2%) had a training/internship/clinical rotation in psychiatry previously. Participants’ general demographic and baseline characteristics are presented in **Table 1**.

**Table 1:** General demographic and baseline characteristics of participants.

Demographics	n	%
Gender		
Male	114	28.8
Female	282	71.2
Prefer to self-describe	0	0
Age group		
18-25 years old	226	57.1
26-35 years old	83	21
36-45 years old	77	19.4
46-55 years old	9	2.3
55 or older	1	0.3
Marital status		
Married/in a relationship	125	31.6
Single	271	68.4
Education level		
Middle school	4	1
High school	244	61.6
University	118	29.8
Higher education	30	7.6
Occupation		
Doctor	25	6.3
Nurse	133	33.6
Other allied health personnel	238	60.1
History of psychiatric treatment		
Yes	21	5.3
No	375	94.7
Having a relative with mental illness		
Yes	85	21.5
Relationship		
Parent	23	27.4
Siblings	6	7.1
Child	1	1.2
Spouse	3	3.6
Other	51	60.7
Diagnosis		
Alcohol use disorder	4	4.8
Schizophrenia	10	11.9
Depression	38	45.2
Other	32	38.1
No	311	78.5
If present, source of information about the illness of oneself or relative		
Education	25	27.5
Television	2	2.2
Social media	4	4.4
Internet	12	13.2
Other	48	52.7
Previous training/internship/rotation in psychiatry services?		
Yes	84	21.2
No	312	78.8

‘n’ represents ‘number’ and the ‘%’ symbol represents ‘percentage’.

Participants’ evaluation and responses of the two case scenarios regarding substance and alcohol use disorders are presented in **Table 2**. Although there was no significant difference between the proportions of participants who described the cases as a physical (p=0.082) or psychiatric illness (p=0.290), significantly more participants stated that the substance use scenario was describing an illness (86.2% vs. 72.9%, p<0.001). Less than one-fourth of participants (23.3%) reported that the substance use scenario was not an illness, whereas 30.7% reported the same for the alcohol use disorder case (p=0.063). Regarding the etiology of the illness, the proportion of participants associating the case with previous/existing socioeconomic problems was 82.3% for alcohol use, and 74.8% for substance use (p=0.023). Approximately half of the study group (50.5%) thought anyone could experience SUD, while 63.7% of the participants mentioned anyone could experience AUD (p=0.001). However, more participants believed that SUD was a social problem (78.8% vs. 71.6%, p=0.029). While evaluating participants’ social distances, higher proportions were observed for participants thinking that the SUD cases should not freely roam in the community (54.8% vs. 44.7%, p=0.014), participants would be more reluctant to marry someone with SUD (19.5% vs. 11.8%, p=0.007), would be less concerned about an AUD diagnosed neighbor (29.8% vs. 21.8%, p=0.023), would not rent a property to a person with SUD (67.2% vs. 56.4%, p=0.006), perceived people with SUD as aggressive (72.5% vs. 64.9%, p=0.05), and that people with SUD cannot make appropriate decisions in their daily life (85.1% vs. 77.2%, p=0.011). Regarding treatment-related views, a higher proportion of the study population reported that SUD cases could be treated with pharmacotherapy (80.3% vs. 71.7%, p=0.028), medicines used to treat SUD may have severe adverse effects (77.8% vs. 62.9%, p=0.005), and SUD cases could not be cured completely (24.7% vs. 15.2%, p=0.003).

**Table 2:** Participants’ considerations about the clinical case-scenarios.

Variable	Substance use disorder	Alcohol use disorder	p
	n (%)	n (%)	
Describing the illness			
Text describes a physical illness	75 (26.3)	59 (20.2)	0.082
Text describes a psychiatric illness	277 (82.4)	255 (79.2)	0.290
Text describes an illness	293(86.2)	229 (72.9)	**<0.001**
Text describes no illness	60 (23.3)	77 (30.7)	0.063
Describing the etiology of the illness			
It is associated with personality structure	191 (65.9)	194 (71.9)	0.127
It is associated with social problems (such as unemployment, poverty, family problems)	235 (74.8)	256 (82.3)	**0.023**
Everyone can experience such a situation	163 (50.5)	211 (63.7)	**0.001**
It is a social problem	272 (78.8)	234 (71.6)	**0.029**
It is a moral problem	114 (43)	119 (43.6)	0.894
These people have a genetic tendency	83 (42.1)	95 (43.8)	0.735
It is a mental weakness	292 (84.6)	260 (79.3)	0.070
Social distance			
Should not freely roam in the community	164 (54.8)	132 (44.7)	**0.014**
I can work together	79 (27)	89 (29.8)	0.449
I can marry this person	40 (11.8)	61 (19.5)	**0.007**
It does not bother me if this person is my neighbor	69 (21.8)	92 (29.8)	**0.023**
I do not rent my home to this person	215 (67.2)	168 (56.4)	**0.006**
They are aggressive	206 (72.5)	187 (64.9)	**0.050**
They cannot make the right decisions about their lives	285 (85.1)	237 (77.2)	**0.011**
Treatment-related thoughts			
Change of neighborhood (such as going vacation) is important	239 (75.2)	215 (68.3)	0.054
Witch doctors can heal this illness	58 (17.3)	45 (13.4)	0.169
It can be treated by psychotherapy	240 (80.5)	235 (81.9)	0.677
It can be treated by pharmacotherapy	200 (80.3)	165 (71.7)	0.028
Attitudes regarding pharmacotherapy			
Medicines may be addictive	160 (74.8)	119 (70)	0.298
Medicines may have severe adverse effects	130 (77.8)	83 (62.9)	**0.005**
Thoughts regarding prognosis			
It does not resolve completely	79 (24.7)	49 (15.2)	**0.003**
This illness does not heal unless social problems once solved	155 (55.2)	137 (48.8)	0.129
It is a curable illness	316 (89.8)	300 (88.8)	0.667

Values presented in bold indicate statistical significance (p < 0.05).

‘n’ represents ‘number’ and the ‘%’ symbol represents ‘percentage’.

The evaluation of participants’ general attitudes about “what people with addiction should do” found a higher proportion stating that individuals with a SUD should go to a doctor (59.8% vs. 27.3%), and people with SUD should improve their living conditions (8.1% vs. 4%). When participants were asked what they would do if they had similar symptoms, more participants responded that they would see a doctor if they had SUD (87.9% vs. 81.8%). The clinical department that individuals with ASUD should visit was identified as the psychiatry department for both case scenarios (**Table 3**).

**Table 3:** Participants’ general attitudes about what the individuals in the vignettes should do.

Variable	Substance use disorder	Alcohol use disorder	p
	n (%)	n (%)	
What should the case do?			0.173
Go to a doctor	237 (59.8)	277 (27.3)	
Go on a vacation	20 (5.1)	13 (3.3)	
Should be strong	120 (30.3)	122 (30.8)	
Should improve the living conditions	16 (4)	32 (8.1)	
Should take traditional treatment	3 (0.8)	2 (0.5)	
What would you do in such a case?			0.152
I would go to a doctor	348 (87.9)	324 (81.8)	
I would go on a vacation	24 (6.1)	39 (9.8)	
I would seek spiritual support	10 (2.5)	12 (3)	
I would seek alternative treatments	10 (2.5)	12 (3)	
I would do nothing	4 (1)	9 (2.3)	
Which specialist should the case go to?			
Family physician	31 (7.8)	34 (8.6)	0.003
Psychiatrist	205 (51.8)	213 (53.8)	
Internal medicine specialist	6 (1.5)	20 (5.1)	
Should not go to a doctor	6 (1.5)	15 (3.8)	
Which specialist should you go to in such a case?			0.133
Family physician	18 (4.5)	28 (7.1)	
Psychiatrist	235 (59.3)	230 (58.2)	
Internal medicine specialist	6 (1.5)	15 (3.8)	
Neurologist	3 (0.8)	3 (0.8)	

‘n’ represents ‘number’ and the ‘%’ symbol represents ‘percentage’.

## 4 Discussion

Our vignette-based study evaluated the stigma patterns of ASUD cases among healthcare workers in a tertiary health-care facility; to the best of our knowledge, this is the first study to assess the possible stigmatization differences between substance use disorder and alcohol use disorder in health professionals. Additional strengths of this study include the evaluation of possible factors affecting the differences in stigmatization patterns, such as attendants’ familiarity with any mental illness, aspects of origin, and treatment methodologies for the two conditions.

The stigma of individuals with substance use disorders is a two-sided phenomenon, resulting in various unfavorable consequences. While previous research suggests that stigma and the associated attitudes of health professionals toward individuals with substance use disorders cause delays in treatment or interruptions in relapsed cases (13, 14), there is also a tendency among ASUD patients to develop self-stigma or a loss of self-esteem (13). Thus, stigma among healthcare workers should be evaluated in detail to prevent barriers to patient treatment. According to a recent review (15), 20% to 51% of health professionals might have disfavorable beliefs about ASUD cases, 46% of emergency physicians thought people with AUD were dangerous, while 60% of emergency physicians thought SUD patients were dangerous, and health professionals tended to have negative professional attitudes towards patients. Our study also revealed that participants believed patients with SUD were more aggressive and less capable to make reasonable decisions than patients with AUD. McLaughlin et al. (16) evaluated health and social care professionals’ perceptions and experiences of substance users in Northern Ireland, reporting that participants, including nurses, general practitioners, health visitors, pharmacists, social and health promotion workers, and health center managers, all had strong opposing views towards SUD patients, to the extent of considering not to provide care for those individuals, or not to refer them to other professionals specializing in ASUD. Evaluating nurses’ therapeutic attitudes towards patients with SUD, Ford et al. (17) found that this occupational group had poor motivation and satisfaction with the care they provided to people diagnosed with SUD. It must be emphasized that providing health services to people with ASUD is not limited to providing care for their mental and behavioral disturbances. In fact, patients with ASUD primarily seek healthcare for various problems or complaints unrelated to ASUD (18). Previous studies showed that slightly less than one-fourth of patients with an AUD seek treatment during their lifetime, and only 15% of patients with SUD apply for medical care within a calendar year (19, 20). These patients are generally admitted to various departments in a healthcare facility, and tend not to mention their substance use problems due to possible stigmatization patterns (8). From this perspective, we constituted a research population of diverse occupational status in a tertiary healthcare center. Our results suggest that among healthcare providers, the stigma for SUD cases was higher in some aspects than for patients with AUD.

The participants’ assessments of the case vignettes showed that more than 60% of the participants accepted ASUD cases as constituting a social concern; ASUD was associated with personality structure and mental weakness, and ASUD is triggered by socioeconomic difficulties (unemployment, poverty, family matters, etc.). These findings may be interpreted as a tendency among participants to accept ASUD associated with the “moral model”. Additionally, unlike SUD, AUD is considered a condition that anyone can experience, mainly when socioeconomic difficulties are present. More participants defined SUD as a “disease” than AUD. This can be interpreted as participants having less stigma towards AUD cases, based on the moral model acceptance of the disorder. An earlier Turkish study also detected more negative attitudes towards SUD compared to AUD among non-psychiatric physicians (21). This was also partly evident in our results, in that AUD was considered a social problem-triggered disorder that could be experienced by anyone. However, a more severe discriminative perception was attributed to SUD, which was regarded as a moral fault of the patient. These attributional differences were further emphasized by the statement that SUD cases should not roam freely in the community, a sentiment less frequently recognized for AUD cases. Since it has been postulated that the perception of the model influences the attitudes of health professionals towards ASUD patients (15), the findings of our study also indicate possible interactions between the etiological perception of ASUD, and healthcare workers’ attitudes.

No previous study has focused directly on the possible differences in stigmatization patterns between AUD and SUD cases, but several studies have inferential findings. Some studies found substance use to be more associated with stigmatization, secondary to its perceptions such as being more irreversible and socially problematic than alcohol use disorder (22). According to a recently published study by Parish and colleagues conducted among primary care physicians, emergency medicine physicians, and dentists, stimulant use disorder was found to be the most highly stigmatized condition, followed by opioid and alcohol use disorders. Among the professions, emergency medicine physicians demonstrated the highest stigma scores (23). In a study conducted among healthcare professionals with different occupational roles, participants over the age of 30 were found to exhibit greater social distancing toward individuals with alcohol use disorder, whereas participants under the age of 30 demonstrated greater social distancing toward those with polysubstance use disorder. The same study also reported that healthcare professionals in different roles expressed varying levels of fear toward different substances (24). The current findings corroborate such earlier results, while adding that SUD carries additional layers of stigma.

Our results also provide key information regarding the preventive measures to be taken against the potential barriers of treatment. The most important of these is the unbalanced but present stigma for both disorders. To address this problem, the components of these stigmas must be identified. Apart from the participants’ thoughts on the basis and etiology, beliefs about treatment must also be evaluated. For instance, significantly more participants believed that pharmacotherapy was needed for SUD cases, and that medications might have severe adverse effects. In addition, the proportion of participants who agreed that SUD would not completely recover was significantly higher. Policymakers and institutional in-service training administrators should consider these prejudices when delivering more appropriate healthcare to patients with ASUD. The results of this study align with those of other studies highlighting the importance of emphasizing the disease model of ASUD, changing negative beliefs, and the need to train all healthcare professionals (15).

Despite the study’s valuable results, its various limitations must also be noted. First, it evaluated participants’ perceptions of fictitious case vignettes. This may have affected the responses obtained, and may not entirely reflect practice patterns or in-hospital attitudes towards patients. However, the use of vignettes is a favorable method in various aspects (22). Second, we did not conduct sensitivity analyses or comparisons between demographic subgroups of participants of different ages, years of education, income, occupation, etc., to identify any differences in stigma patterns. Third, all substances listed in the diagnostic systems were presented together in a single vignette, it was not possible to perform separate comparisons for each individual substance. Nevertheless, the study’s strengths make our findings valuable to readers. First, this study is the first to evaluate and compare the stigma associated with alcohol and substance use disorders in a tertiary healthcare setting. Second, the study was conducted in a major healthcare facility in Istanbul, which serves a significant number of patients in the region. Keeping these limitations and strengths in mind, we believe that this study will provide a beneficial inference regarding stigmatization of substance and alcohol use disorders in health settings.

## 5 Conclusion

This study is the first of its kind to be conducted among healthcare workers to evaluate and compare stigma towards alcohol and substance use disorders. Our results showed that stigmatization was present towards these patients in every occupational group working in a major healthcare facility. The stigma patterns were similar to those previously reported in scientific research on the topic, which might suggest an unfavorable effect on healthcare delivery to these patients. The outcomes presented here can guide future studies to thoroughly evaluate the determinants of this established stigma, or can be used as a basis for administrative organizations’ improvement efforts. We further suggest that these results can be fortified by the outcomes of studies conducted to evaluate the unmet clinical needs of patients with substance and alcohol use disorders.

The authors declare that they have no known competing financial interests or personal relationships that could have appeared to influence the work reported in this paper.

## Data Availability

The raw data supporting the conclusions of this article will be made available by the authors, without undue reservation.
