# Supplementary material for: A comparative appraisal of stigma among healthcare workers towards alcohol and substance use disorders: a case vignette study
Source: Front Psychiatry. 2026 Feb 18;17:1634817. doi: 10.3389/fpsyt.2026.1634817 (PMC12956808; doi:10.3389/fpsyt.2026.1634817)
Supplement: Supplementary file 1 [file Table1.docx]

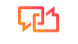
 [Translated from Turkish to English - www.onlinedoctranslator.com](https://www.onlinedoctranslator.com/en/?utm_source=onlinedoctranslator&utm_medium=docx&utm_campaign=attribution)

Dear Participant,

This research is being conducted within the responsibility of Onat YILMAZ, a specialist doctor working at the Psychiatry Clinic of Okmeydanı Education and Research Hospital of Health Sciences University, and Asil BUDAKLI, a specialist doctor working at the Psychiatry Clinic of Bahcesehir University. The aim of this study is to investigate perspectives of healthcare workers about patients with a diagnosis of alcohol or substance use disorder. For this purpose, you are asked to answer the questions below. There are no right or wrong answers to these questions. Please read the instructions at the top of the page carefully, try to give the most appropriate answer and do not leave blank as much as possible. Your answers will be kept confidential by the researchers and will be used for scientific purposes only. Since the answers will be evaluated within the framework of all participants, no identity information is requested from you. Your sincere answers will make the greatest contribution to this study.

Participating in the study is voluntary, however, if you feel uncomfortable for any reason during participation, you have the right to withdraw from the study.

This survey consists of two parts. The first part includes questions prepared to obtain basic sociodemographic information and to determine the status of knowledge about mental illnesses; the second part consists of questions prepared to determine your views and thoughts about the cases presented.

In order to reach objective results and contribute to scientific literature, participants are asked to answer the questions frankly. Please do not write your name or personal information on the papers you have been given.

Thank you in advance for your interest and participation.

PART 1

PERSONAL INFORMATION FORM

1. How do you prefer to identify your gender:

Female ( ) Male ( ) Prefer to self-describe ( )

2. Your Marital Status:

In a relationship/Married ( ) Single ( )

3. Your age:

18-25 ( ) 26-35 ( ) 36-45 ( )

46-55( ) 55 and above ( )

4. The school you graduated from:

Middle School ( ) High School ( ) University/College ( ) Further education ( )

5.Your profession:

Physician ( ) Nurse ( )

Other Health class ( ) Non-health ( )

6. Do you have a history of treatment about mental health :

Yes ( ) No ( )

7. Does any of your contacts have a history of treatment about mental health:

There is ( ) No ( )

8. The level of relationship of your contact with a history of treatment for mental illness:

Parents ( ) Brother ( )

Child ( ) Spouse( ) Other ( )

9. Diagnosis of your relative with mental illness is:

Substance use ( ) Alcohol use ( ) Schizophrenia ( ) Depression ( )

Other ( )

10.Where did you get information about this disease?

During education ( ) Television ( )

Social media ( ) Internet ( )

Other ( )

11. Have you had a education about Mental Health/Psychiatry?

Yes ( ) No ( )

PART 2

In this section, you are asked to answer questions using various case examples to learn your views on mental illness and psychiatric illness. Please answer these questions according to your own thoughts;

**1-Strongly Disagree, 2-Disagree, 3-No Opinion, 4-Agree, 5-Strongly Agree,**tick one of the options **( X )** Answer by putting.

**CASE EXAMPLE 1**

Mrs A is a 27-year-old married housewife with 2 children. Mrs A often uses substances (like marijuana, cannabis, opioid or cocaine) in larger amounts or for longer periods than desired, makes constant or unsuccessful efforts to quit or control it, and continues to use substances (like marijuana, cannabis, opioid or cocaine) despite experiencing social or interpersonal problems caused or increased by the effects of substance use.

| QUESTION NO | QUESTION | **I STRONGLY DISAGREE** | **I DON'T AGREE** | **I HAVE NO IDEA** | **I AGREE** | **I ABSOLUTELY AGREE** |
| --- | --- | --- | --- | --- | --- | --- |
|  | Mrs A has a physical illness |  |  |  |  |  |
|  | Mrs A has a Mental Illness |  |  |  |  |  |
|  | This situation of Mrs A stems from her personality structure. |  |  |  |  |  |
|  | Condition of Mrs A is due to the social problems she is experiencing (such as unemployment, poverty, family problems). |  |  |  |  |  |
|  | Mrs A does not have any illness. |  |  |  |  |  |
|  | Anyone can experience the condition that Mrs A experiences |  |  |  |  |  |
|  | Substance use is a social problem |  |  |  |  |  |
|  | Patients with substance use disorder would not completely recover |  |  |  |  |  |
|  | Changing the environment (going on vacation) is important |  |  |  |  |  |
|  | Pilgrims or muslim preachers can cure this disease |  |  |  |  |  |
|  | This disorder becomes evident only due to social problems |  |  |  |  |  |
|  | This disorder will not heal unless social problems are resolved. |  |  |  |  |  |
|  | Patients with substance use disorder should not roam freely in society. |  |  |  |  |  |
|  | I can work with someone who has substance use disorder. |  |  |  |  |  |
|  | I can marry someone with a diagnosis of substance use disorder. |  |  |  |  |  |
|  | It doesn't bother me if my neighbour is a person with a diagnosis of substance use disorder. |  |  |  |  |  |
|  | I would not rent my house to someone who is a person with a diagnosis of substance use disorder. |  |  |  |  |  |
|  | People with a diagnosis of substance use are aggressive |  |  |  |  |  |
|  | Substance use disorder is a moral issue |  |  |  |  |  |
|  | People with a diagnosis of substance use disorder cannot make right decisions about their lives. |  |  |  |  |  |
|  | Substance use disorder is a disease |  |  |  |  |  |
|  | Substance use disorder is a disease that can be treated with medication. |  |  |  |  |  |
|  | Substance use disorder can be treated with psychotherapy (talking therapy) |  |  |  |  |  |
|  | Treatments used in substance use disorder might cause addiction |  |  |  |  |  |
|  | Treatments used in substance use disorder have serious side effects |  |  |  |  |  |
|  | There is a genetic predisposition in patients with substance use disorder |  |  |  |  |  |
|  | Substance use disorder is a state of mental weakness |  |  |  |  |  |

**1. Mrs A, to get rid of this situation:**

1. Firstly, Mrs A needs to see a doctor.
2. Firstly, Mrs A needs to be strong, Mrs A can overcome this condition if motivated.
3. First of all, Mrs A needs to improve environmental conditions.
4. Firstly, Mrs A needs to go on a vacation and get away from current environment.
5. Firstly, Mrs A should choose traditional methods.

**2. If Mrs A wants to go to the doctor;**

1. Should go to the family physician
2. Should go to an internal medicine (internal diseases specialist) doctor.
3. Should go to a neurologist.
4. Should go to a psychiatrist (mental health specialist).
5. There is no need to go to the doctor

**3. What would you do if you had these complaints/this condition?**

1. Firstly, I would go on a vacation
2. Firstly, I would go to the doctor
3. Firstly, I would seek for religious support
4. Firstly, I would prefer traditional methods (acupuncture, cupping, hirudo etc.)
5. I wouldn't do anything

**4. If you decide to go to ta doctor, which of the following would you apply first?**

1. Family physician
2. Psychiatrist/Mental health specialist
3. Internal medicine specialist
4. Doctor of Neurology

**CASE EXAMPLE 2**

Mrs A is a 27-year-old married housewife with 2 children. Mrs A often uses alcohol in larger amounts or for longer periods than desired, makes constant or unsuccessful efforts to quit or control it, and continues to use alcohol despite experiencing social or interpersonal problems caused or increased by the effects of alcohol.

| QUESTION NO | QUESTION | **I STRONGLY DISAGREE** | **I DON'T AGREE** | **I HAVE NO IDEA** | **I AGREE** | **I ABSOLUTELY AGREE** |
| --- | --- | --- | --- | --- | --- | --- |
|  | Mrs A has a physical illness |  |  |  |  |  |
|  | Mrs A has a Mental Illness |  |  |  |  |  |
|  | This situation of Mrs A stems from her personality structure. |  |  |  |  |  |
|  | Condition of Mrs A is due to the social problems she is experiencing (such as unemployment, poverty, family problems). |  |  |  |  |  |
|  | Mrs A does not have any illness. |  |  |  |  |  |
|  | Anyone can experience the condition that Mrs A experiences |  |  |  |  |  |
|  | Alcohol use disorder is a social problem |  |  |  |  |  |
|  | Patients with alcohol use disorder would not completely recover |  |  |  |  |  |
|  | Changing the environment (going on vacation) is important |  |  |  |  |  |
|  | Pilgrims or muslim preachers can cure alcohol use disorder |  |  |  |  |  |
|  | This disorder becomes evident only due to social problems |  |  |  |  |  |
|  | This disorder will not heal unless social problems are resolved. |  |  |  |  |  |
|  | Patients with alcohol use disorder should not roam freely in society. |  |  |  |  |  |
|  | I can work with someone with a diagnosis of alcohol use disorder |  |  |  |  |  |
|  | I can marry someone who has a dagnosis of alcohol use disorder. |  |  |  |  |  |
|  | It doesn't bother me if my neighbour is a person with a diagnosis of alcohol use disorder. |  |  |  |  |  |
|  | I would not rent my house to someone who is a person with a diagnosis of alcohol use disorder. |  |  |  |  |  |
|  | People with a diagnosis of alcohol use disorder are aggressive |  |  |  |  |  |
|  | Alcohol use disorder is a moral issue |  |  |  |  |  |
|  | People with a diagnosis of alcohol use disorder cannot make tright decisions about their lives. |  |  |  |  |  |
|  | Alcohol use disorder is a disease |  |  |  |  |  |
|  | Alcohol use disorder is a disease that can be treated with medication. |  |  |  |  |  |
|  | Alcohol use disorder can be treated with psychotherapy (talking therapy) |  |  |  |  |  |
|  | Treatments used in alcohol use disorder might cause addiction |  |  |  |  |  |
|  | Treatments used in alcohol use disorder have serious side effects |  |  |  |  |  |
|  | There is a genetic predisposition in patients with alcohol use disorder |  |  |  |  |  |
|  | Alcohol use disorder is a state of mental weakness |  |  |  |  |  |

**1. Mrs A, to get rid of this situation:**

1. Firstly, Mrs A needs to see a doctor.
2. Firstly, Mrs A needs to be strong, Mrs A can overcome this condition if motivated.
3. First of all, Mrs A needs to improve environmental conditions.
4. Firstly, Mrs A needs to go on a vacation and get away from current environment.
5. Firstly, Mrs A should choose traditional methods.

**2. If Mrs A wants to go to the doctor;**

1. Should go to the family physician
2. Should go to an internal medicine (internal diseases specialist) doctor.
3. Should go to a neurologist.
4. Should go to a psychiatrist (mental health specialist).
5. There is no need to go to the doctor

**3. What would you do if you had these complaints/this condition?**

1. Firstly, I would go on a vacation
2. Firstly, I would go to the doctor
3. Firstly, I would seek for religious support
4. Firstly, I would prefer traditional methods (acupuncture, cupping, hirudo etc.)
5. I wouldn't do anything

**4. If you decide to go to ta doctor, which of the following would you apply first?**

1. Family physician
2. Psychiatrist/Mental health specialist
3. Internal medicine specialist
4. Doctor of Neurology
